# Effect of body composition on the athletic performance of soccer referees

**DOI:** 10.1017/jns.2023.47

**Published:** 2023-06-27

**Authors:** Azad Ilhan, Surhat Muniroglu, Neslişah Rakıcıoğlu

**Affiliations:** 1 Faculty of Health Sciences, Department of Nutrition and Dietetics, Hacettepe University, Sıhhıye 06100, Ankara, Turkey; 2 Faculty of Sports Sciences, Department of Coaching Education, Ankara University, Ankara 06830, Turkey

**Keywords:** Athletic performance, Body composition, Nutritional status, Soccer referees, ACSM, American College of Sports Medicine, BMI, body mass index, FM, fat mass, FFM, fat-free mass, IOC, International Olympic Committee, RDA, recommended dietary allowance, UEFA, Union of European Football Associations, WC, waist circumference

## Abstract

Nutrition plays an important role in improving sports performance. The present study aimed at nutritional assessment and examined the relationship between athletic performance and body composition in soccer referees at different levels. The study participants were 120 male soccer referees. 5, 10 and 30 metres (m) sprint tests to measure speed and cooper test for physical fitness were applied in the referees. Participants were divided into two groups as city and class soccer referee. The anthropometric measurements, excluding fat mass (FM) (%), were higher in class referees. Fat mass (%) differences (14⋅1 ± 4⋅28 *v.* 12⋅3 ± 4⋅41) were statistically significant (*P* < 0⋅05). Daily energy and nutrient intakes were similar. The inadequacy percentages of energy, vitamin A and calcium were the highest (29⋅2, 30⋅0 and 34⋅2 %, respectively). It was found that a negative significant correlation between FM% and cooper test score (*P* < 0⋅01; *r* = −0⋅35), a positive significant correlation between FM% and 5, 10 and 30 m sprint test scores (*P* < 0⋅01, *r* = 0⋅38; *P* < 0⋅01, *r* = 0⋅38 and *P* < 0⋅01, *r* = 0⋅48, respectively). Similarly, there was a negative significant correlation between waist circumference (WC) and cooper test score (*P* < 0⋅01; *r* = −0⋅31), a positive significant correlation between WC and 5, 10 and 30 m sprint test scores (*P* < 0⋅01, *r* = 0⋅33; *P* < 0⋅01, *r* = 0⋅40; *P* < 0⋅01, *r* = 0⋅33, respectively). Nutritional recommendations for soccer referees should be made specific to the individual, considering body composition, training intensity and match frequency by a dietician.

## Introduction

Soccer referees are a specific group of athletes whose careers reach the highest level between 30 and 45^(1)^. They are considered athletes because following regular and intensive training programmes to have a high level of performance^(2)^. They must have excellent refereeing knowledge, good communication skills and ensure good emotional control for successful match management^(3)^. As with all other sports, referees must have an optimal fitness level to perform a good performance^(4)^.

For elite athletes, nutritional strategies have become important in their physical condition and training. The referee's performance is influenced by factors such as physical training, eating behaviours and body composition^(1)^. Diet quality affects body composition^(5)^. To maintain and improve their performance, soccer referees are recommended to consume a high-quality diet, to have optimal body composition. Determining the required intake amounts in terms of energy, macro and micronutrients is essential. A limited number of studies examine soccer referees' nutritional behaviour. Teixeira *et al.*^(6)^ found that the referees’ carbohydrate intake was below the recommended level and they were malnourished in terms of micronutrients. Another study stated that dietary energy from carbohydrates was not sufficient when considering their work^(4)^.

Soccer referees have a maximum oxygen uptake of 51⋅9 ml/kg/min and perform similarly to soccer players covering a distance of approximately 10 000–13 000 metres (m) throughout the match^(7,8)^. While there are guidelines^(9)^ for athlete nutrition, there is no guideline for soccer referees. Referees who are on average 15 years older than elite athletes should not be ignored when it comes to proper nutrition recommendations. Today, elite soccer referees are given nutritional recommendations similar to those of soccer players, regardless of differences in age, type of activity, body composition and physical condition^(10)^. Optimal body composition enables athletes to move more effectively during training and competitions^(11,12)^. Therefore, it is important to assess the nutritional status and determine the relationship between body composition and athletic performance in soccer referees. The present study aimed at nutritional assessment and examined the relationship between athletic performance and body composition in soccer referees at different levels.

## Materials and methods

### Data collection

The study participants were 120 active male soccer referees (between the ages 18 and 47) selected by the random sampling method from 250 soccer referees recruited from Turkey Football Federation living in Ankara. The referees on a special diet and not actively involved in the matches were excluded from the study. According to the level of refereeing, participants were classified as city and class referees from low to high. The match in which the referee's control is classified as follows: city referees at the provincial level and class referees at the national level.

The general characteristics of subjects (such as age, educational status, classification and years of refereeing experience) and nutritional habits were recorded with the help of a questionnaire.

### Anthropometric measurements

Anthropometric measurements (body weight and height, fat mass (FM), waist circumference (WC) and hip circumference) were performed while subjects wearing underwear in a fasting state (12 h). Body weight and FM were measured using a multi-frequency bioelectrical impedance analyzer (TANITA-MC980, Japan, 0⋅1 kg accuracy)^(13)^. Height, WC and hip circumference were measured by the researchers according to proper methods^(14)^. Body mass index (BMI) was calculated as weight/height^2^.

### Energy and nutrients intake of the soccer referees

Food consumption was assessed through a 24-hour recall method. The dietary recalls were performed through face-to-face interviews and a trained dietitian. A Turkish food photograph catalog was used to assist respondents in identifying the actual quantity of the foods^(15)^. Standard dishes description was used to estimate the amounts of food in one portion consumed outside the institutions^(16)^. Gram amounts of nutrients consumed were entered into the BEBIS-8 (Nutrition Information Systems Software) computer program and daily energy and other nutrients were analysed^(17)^. Nutrient intake below two-thirds of the Recommended Dietary Allowance (RDA) (<67 %) was considered low^(18)^.

### Assessment of the athletic performance level

The soccer referees performed 5, 10 and 30 m sprint tests to measure speed and cooper test for physical fitness. Before the tests, all referees participated in a 20-minute warm-up workout. The sprint tests were practiced on the synthetic turf and performed twice. During the waiting times between tests, participants were kept active by jumping rope and stretching exercises to avoid cooling down. Sprint tests have similar protocols. The photocells settled the starting and finishing points of the distances 5, 10 and 30 m. All referees started from 1 m behind the starting photocell. The best scores of the referees were recorded.

The cooper test^(19)^ was conducted at the 400 m synthetic athletics track. The referees ran the maximum distance they could cover in 12 min by the test protocol. The covered distance was measured with the help of markers located at 50 m intervals on the runway^(20)^.

### Statistical analysis

SPSS (version 23; IBM, Armonk, NY) package program was used for statistical analysis. Data were analysed via Kolmogorov–Smirnov or Shapiro–Wilks tests. Descriptive statistics were expressed using mean and standard deviation (mean ± sd) for continuous variables while frequency and percentages (%) for categorical variables. The significance was set at *P* < 0⋅05.

## Results

The participants’ general characteristics were grouped according to referee classification and shown in Table 1. The sample of the study consisted of 120 male soccer referees in different classifications (69 city and 51 class referee) with a mean age of 26⋅1 ± 4⋅51. The refereeing time was 4⋅51 ± 3⋅73 years for city referees and 7⋅00 ± 3⋅42 years for class referees (*P* < 0⋅05). Only 30⋅0 % of referees studied nutrition education during the education process, and most of them (68⋅3 %) stated that the source of their nutrition knowledge was TV-internet. The mean number of the main meal was higher in class referees than in city referees (2⋅80 ± 0⋅40 *v.* 2⋅49 ± 0⋅50) while the snack meal was higher in city referees than class referees (1⋅17 ± 0⋅92 *v.* 0⋅80 ± 0⋅89) (*P* < 0⋅05). It was determined that 59⋅2 % of referees skipped meals and the most skipped meal was lunch (31⋅7 %).

**Table 1.** General characteristics of subjects according to soccer referee classification

	City referee (*n* 69)		Class referee (*n* 51)		All subjects (*n* 120)	
	*n*	%	*n*	%	*n*	%
Nutrition education						
Yes	18	26⋅1	18	35⋅3	36	30⋅0
No	51	73⋅9	33	64⋅7	84	70⋅0
Sources of nutrition knowledge						
Dietitian	12	17⋅4	11	21⋅6	23	19⋅2
Doctor	3	4⋅3	4	7⋅8	7	5⋅8
TV-internet	50	72⋅5	32	62⋅8	82	68⋅3
Friend	4	5⋅8	4	7⋅8	8	6⋅7
Meal skipping (%)						
Yes	49	71⋅0	22	43⋅1	71	59⋅2
No	20	29⋅0	29	56⋅9	49	40⋅8
Meal skipped (%)						
Breakfast	18	26⋅1	8	15⋅7	26	21⋅7
Lunch	25	36⋅2	13	25⋅5	38	31⋅7
Dinner	6	8⋅7	1	2⋅0	7	5⋅8
Age (year)	25⋅1 ± 4⋅87		27⋅5 ± 3⋅55		26⋅1 ± 4⋅51	
Refereeing time (year)^a^	4⋅51 ± 3⋅73		7⋅00 ± 3⋅42		5⋅57 ± 3⋅79	
Main meal consumption^a^	2⋅49 ± 0⋅50		2⋅80 ± 0⋅40		2⋅63 ± 0⋅49	
Snack meal consumption^a^	1⋅17 ± 0⋅92		0⋅80 ± 0⋅89		1⋅02 ± 0⋅93	

*P*-values calculated with the independent samples *t*-test.

a Mean values were significantly different (*P* < 0⋅05).

The anthropometric measurements according to classification are shown in Table 2. The higher mean values (excluding FM%) were determined in class referees. The differences in fat mass of city and class referees (14⋅1 ± 4⋅28 % *v.* 12⋅3 ± 4⋅41 %) were statistically significant (*P* < 0⋅05).

**Table 2.** Anthropometric measurements of subjects according to soccer referee classification

	City referee (*n* 69)	Class referee (*n* 51)	*P*-value	All subjects (*n* 120)
Anthropometric measurement	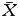 ± sd	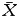 ± sd		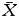 ± sd
Weight (kg)	72⋅9 ± 8⋅60	74⋅8 ± 6⋅47	0⋅17	73⋅7 ± 7⋅79
Height (cm)	178⋅9 ± 5⋅21	180⋅9 ± 4⋅67	0⋅09	179⋅8 ± 5⋅06
Body mass index (BMI) (kg/m^2^)	22⋅73 ± 2⋅13	22⋅84 ± 1⋅66	0⋅76	22⋅77 ± 1⋅94
Fat mass (%)	14⋅1 ± 4⋅28	12⋅3 ± 4⋅41	**0⋅04**	13⋅4 ± 4⋅39
Waist circumference (cm)	83⋅2 ± 6⋅43	84⋅5 ± 4⋅31	0⋅24	83⋅7 ± 5⋅73
Hip circumference (cm)	100⋅5 ± 4⋅66	101⋅2 ± 3⋅78	0⋅41	100⋅7 ± 4⋅34
Waist-hip ratio	0⋅82 ± 0⋅04	0⋅83 ± 0⋅03	0⋅28	0⋅83 ± 0⋅04

Bold type indicates statistical significance (*P* < 0.05).

*P*-values calculated with the independent samples *t*-test.

Table 3 shows the referees’ daily energy and nutrient intake and RDA (%). The mean total energy intake of referees was 2244 ± 734⋅19 kcal (2205 ± 649⋅09 and 2297 ± 839⋅59 kcal for city and class referees, respectively). It was found that 29⋅2 % of referees’ energy intakes were not meeting the recommended daily intake levels. Daily energy and nutrient intakes were similar (*P* > 0⋅05). Vitamin A and calcium were the nutrients in which recommended daily intake level was not met at the highest level (30⋅0 and 34⋅2 %, respectively). Carbohydrate, protein and fat intakes per body weight (kg) were determined as 3⋅51 ± 1⋅38, 1⋅23 ± 0⋅49 and 1⋅27 ± 0⋅57 g, respectively.

**Table 3.** Daily energy and nutrient intake, RDA (%) of the subjects

Energy and nutrients	City referee (*n* 69)	Class referee (*n* 51)	*P*-value	All subjects (*n* 120)
Carbohydrate (g)	258⋅60 ± 104⋅61	251⋅73 ± 87⋅95	0⋅70	255⋅68 ± 97⋅54
Carbohydrate (g/kg)	3⋅59 ± 1⋅47	3⋅39 ± 1⋅25	0⋅42	3⋅51 ± 1⋅38
Carbohydrate (%)	47⋅46 ± 8⋅97	45⋅63 ± 8⋅11	0⋅25	46⋅68 ± 8⋅63
Protein (g)	84⋅57 ± 25⋅93	96⋅20 ± 40⋅49	0⋅07	89⋅52 ± 33⋅26
Protein (g/kg)	1⋅18 ± 0⋅41	1⋅30 ± 0⋅58	0⋅21	1⋅23 ± 0⋅49
Protein (%)	16⋅07 ± 3⋅72	17⋅37 ± 5⋅32	0⋅14	16⋅63 ± 4⋅50
Fat (g)	89⋅42 ± 32⋅06	97⋅06 ± 47⋅39	0⋅32	92⋅66 ± 39⋅31
Fat (g/kg)	1⋅25 ± 0⋅48	1⋅31 ± 0⋅68	0⋅57	1⋅27 ± 0⋅57
Fat (%)	36⋅30 ± 8⋅52	36⋅59 ± 8⋅06	0⋅85	36⋅43 ± 8⋅29
Energy (kcal)	2205⋅13 ± 649⋅09	2297⋅39 ± 839⋅59	0⋅52	2244⋅34 ± 734⋅19
RDA (%)	82⋅59 ± 24⋅31	86⋅04 ± 31⋅45		84⋅06 ± 27⋅50
<RDA	19 (27⋅5)	16 (31⋅4)		35 (29⋅2)
Fibre (g)	25⋅89 ± 10⋅63	24⋅31 ± 8⋅25	0⋅38	25⋅22 ± 9⋅68
RDA (%)	89⋅28 ± 36⋅64	83⋅81 ± 28⋅45		86⋅96 ± 33⋅38
<RDA	16 (23⋅2)	18 (35⋅3)		34 (28⋅3)
Vitamin A (μg)	1820⋅08 ± 4115⋅49	1075⋅34 ± 661⋅73	0⋅20	1503⋅57 ± 3162⋅13
RDA (%)	202⋅23 ± 457⋅27	119⋅48 ± 73⋅53		167⋅06 ± 351⋅35
<RDA	22 (31⋅9)	14 (27⋅5)		36 (30⋅0)
Vitamin E (mg)	20⋅42 ± 11⋅59	18⋅78 ± 10⋅68	0⋅43	19⋅72 ± 11⋅20
RDA (%)	136⋅13 ± 77⋅26	125⋅18 ± 71⋅21		131⋅48 ± 74⋅64
<RDA	17 (24⋅6)	12 (23⋅5)		29 (24⋅2)
Vitamin B1 (mg)	1⋅08 ± 0⋅40	1⋅06 ± 0⋅35	0⋅82	1⋅07 ± 0⋅38
RDA (%)	89⋅77 ± 33⋅45	88⋅43 ± 29⋅04		89⋅20 ± 31⋅53
<RDA	18 (26⋅1)	13 (25⋅5)		31 (25⋅8)
Vitamin B2 (mg)	1⋅57 ± 0⋅79	1⋅52 ± 0⋅68	0⋅69	1⋅55 ± 0⋅74
RDA (%)	120⋅76 ± 60⋅78	116⋅65 ± 52⋅59		119⋅01 ± 57⋅25
<RDA	3 (4⋅3)	6 (11⋅8)		9 (7⋅5)
Vitamin B6 (mg)	1⋅45 ± 0⋅53	1⋅58 ± 0⋅73	0⋅29	1⋅51 ± 0⋅63
RDA (%)	111⋅57 ± 40⋅96	121⋅41 ± 56⋅42		115⋅76 ± 48⋅16
<RDA	3 (4⋅3)	7 (13⋅7)		10 (8⋅3)
Vitamin B12 (mg)	6⋅99 ± 12⋅50	5⋅35 ± 3⋅87	0⋅37	6⋅29 ± 9⋅81
RDA (%)	291⋅23 ± 520⋅84	223⋅02 ± 161⋅26		262⋅24 ± 408⋅76
<RDA	5 (7⋅2)	5 (9⋅8)		10 (8⋅3)
Vitamin C (mg)	125⋅92 ± 102⋅57	113⋅01 ± 81⋅03	0⋅46	120⋅43 ± 93⋅87
RDA (%)	139⋅91 ± 113⋅96	125⋅57 ± 90⋅04		133⋅81 ± 104⋅30
<RDA	14 (20⋅3)	10 (19⋅6)		24 (20⋅0)
Calcium (mg)	832⋅42 ± 335⋅07	830⋅96 ± 366⋅43	0⋅98	831⋅80 ± 347⋅24
RDA (%)	83⋅24 ± 33⋅51	83⋅09 ± 36⋅64		83⋅18 ± 34⋅72
<RDA	23 (33⋅3)	18 (35⋅3)		41 (34⋅2)
Magnesium (mg)	339⋅46 ± 108⋅03	355⋅23 ± 170⋅50	0⋅56	346⋅16 ± 137⋅64
RDA (%)	84⋅87 ± 27⋅01	88⋅81 ± 42⋅63		134⋅57 ± 49⋅55
<RDA	15 (21⋅7)	18 (35⋅3)		33 (27⋅5)
Iron (mg)	13⋅37 ± 4⋅99	13⋅57 ± 4⋅96	0⋅82	13⋅46 ± 4⋅96
RDA (%)	133⋅70 ± 49⋅87	135⋅75 ± 49⋅59		134⋅57 ± 49⋅55
<RDA	3 (4⋅3)	2 (3⋅9)		5 (4⋅2)
Zinc (mg)	13⋅07 ± 4⋅93	13⋅37 ± 5⋅34	0⋅74	13⋅20 ± 5⋅09
RDA (%)	118⋅79 ± 44⋅85	121⋅57 ± 48⋅58		119⋅97 ± 46⋅29
<RDA	8 (11⋅6)	3 (5⋅9)		11 (9⋅2)

*P*-values calculated with the independent samples *t*-test. Values indicating the number of referees with food consumption below RDA (<RDA) are shown as numbers and percentages (%) in parentheses.

RDA, recommended daily allowance.

Soccer referees’ athletic test scores are shown in Table 4 and the relationship between these scores and anthropometric measurements is shown in Table 5. The athletic test scores of city referees were 2861 ± 195⋅18 m for the cooper test; 0⋅98 ± 0⋅05, 1⋅71 ± 0⋅07 and 4⋅29 ± 0⋅15 s for the 5, 10 and 30 m sprint tests, respectively. For class referees, these values are 3003 ± 144⋅32 m for the cooper test; 0⋅96 ± 0⋅05, 1⋅70 ± 0⋅07 and 4⋅22 ± 0⋅20 s for the 5, 10 and 30 m sprint tests, respectively. Differences between cooper test scores were statistically significant (*P* < 0⋅001) (Table 4). When all the participants were evaluated, it was found that a negative significant correlation between FM% and cooper test score (*P* < 0⋅01; *r* = −0⋅35), a positive significant correlation between FM% and 5, 10 and 30 m sprint test scores (*P* < 0⋅01, *r* = 0⋅38; *P* < 0⋅01, *r* = 0⋅38 and *P* < 0⋅01, *r* = 0⋅48, respectively). Similarly, there was a negative significant correlation between WC and cooper test score (*P* < 0⋅01; *r* = −0⋅31), a positive significant correlation between WC and 5, 10 and 30 m sprint test scores (*P* < 0⋅01, *r* = 0⋅33; *P* < 0⋅01, *r* = 0⋅40; *P* < 0⋅01, *r* = 0⋅33, respectively). Also, it was found that a positive significant correlation between waist-hip ratio and 5, 10 and 30 m sprint test scores (*P* < 0⋅01, *r* = 0⋅32; *P* < 0⋅01, *r* = 0⋅29 and *P* < 0⋅01, *r* = 0⋅21, respectively) (Table 5).

**Table 4.** Athletic test scores of soccer referees

Athletic test	City referee (*n* 69)		Class referee (*n* 51)		*P*-value
	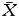 ± sd	Median	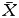 ± sd	Median	
5 m sprint (s)	0⋅98±0⋅05	0⋅98	0⋅96±0⋅05	0⋅97	0⋅06
	(0⋅89–1⋅10)		(0⋅86–1⋅06)		
10 m sprint (s)	1⋅71±0⋅07	1⋅71	1⋅70±0⋅07	1⋅70	0⋅3
	(1⋅59–1⋅88)		(1⋅55–1⋅81)		
30 m sprint (s)	4⋅29±0⋅15	4⋅28	4⋅22±0⋅20	4⋅30	0⋅08
	(3⋅99–4⋅70)		(3⋅82–4⋅65)		
Cooper test (m)	2861⋅3±195⋅18	2850⋅0	3002⋅6±144⋅32	3050	**<0⋅001**
	(2600–3250)		(2700–3200)		

Bold type indicates statistical significance.

*P*-values calculated with the independent samples *t*-test.

The figures in parentheses indicate minimum–maximum values.

**Table 5.** The relationship between the athletic test scores and anthropometric measurements (*r*)

All participants	5 m	10 m	30 m	Cooper test
BMI (kg/m^2^)	0⋅19	0⋅29**	0⋅26**	−0⋅19
Fat mass (%)	0⋅38**	0⋅38**	0⋅48**	−0⋅35**
Waist circumference (cm)	0⋅33**	0⋅4**	0⋅33**	−0⋅31**

Pearson correlation analysis was applied to all groups.

BMI, body mass index.

Bold type indicates statistical significance (**P < 0⋅01.)

## Discussion

Soccer referees with an average refereeing time of 5⋅57 years participated in this study and class referees had longer refereeing time. Considering the importance of experience for success in football refereeing, it is not surprising that the higher level has a longer period of refereeing. Nutrition plays an important role in improving health and sports performance^(2)^. For this reason, athletes are given nutritional strategies as part of physical conditioning and training. Soccer referees are considered athletes because they have regular and intensive training programmes to achieve a high-performance level^(21)^. The International Olympic Committee (IOC) recognises healthy eating and/or good eating habits as the first step in achieving the best performance^(22)^. The consumption of the main meals is important to obtain the energy, macro and micronutrients needed daily. A study showed that professional soccer players were more attentive to consuming the main meals and skipped fewer meals than amateur soccer players^(23)^. Similarly, in the present study, the class referees had a higher number of main meal consumption and lower snack meal consumption (Table 1). These results show that the importance given to food and nutrition increases as the level of athletes increases regardless of the sports branch.

The American College of Sports Medicine (ACSM) stated that the ratio of energy from carbohydrates, protein and fat for athletes should be 55–70, 15–20 and <30 % and 5⋅0–7⋅0, 1⋅2–2⋅0 and 1⋅0 g per body weight (kg), respectively^(9)^. Teixeira *et al.*^(6)^ reported the mean daily energy intake of soccer referees as 2819 ± 279 kcal and the ratios of energy from carbohydrates, proteins and fats were 44⋅4 ± 4⋅4, 18⋅4 ± 1⋅5 and 34⋅6 ± 4⋅1 %, respectively. In the present study, the mean daily energy intake was 2244⋅3 ± 734⋅19 kcal and the ratios of energy from carbohydrates, proteins and fats were 47, 17, and 36 %, respectively (Table 3). Mascherini *et al.*^(1)^ stated that the carbohydrate, protein and fat intakes per body weight (kg) of soccer referees were 3⋅1 ± 0⋅8, 1⋅1 ± 0⋅3 and 1⋅3 ± 0⋅3 g, respectively. In the present study, carbohydrate, protein and fat intakes per body weight (kg) were 3⋅51 ± 1⋅38, 1⋅23 ± 0⋅49 and 1⋅27 ± 0⋅57 g, respectively (Table 3). Both studies show that carbohydrate consumption is below the recommended level and fat consumption is above the recommended level. The most suitable source of energy is carbohydrates. Adequate carbohydrate intake is important for optimal physical performance as it is the key element for the brain, central nervous system and muscles to perform various movements^(24)^. Soccer referees often perform at long-term, continuous or intermittent high intensity. For these types of performance, it is necessary to maintain high carbohydrate levels and avoid depletion of glycogen stores associated with reduced work efficiency, impaired skill and concentration levels^(25)^. Adaptation to high-fat low-carb diets reduces the presence of carbohydrates as a substrate during exercise^(26)^. In addition, the inadequacy percentages of vitamin A and calcium were the highest (30⋅0 and 34⋅2 %, respectively). Vitamin A improves vision and has antioxidant properties^(27)^, while calcium plays an important role in preventing stress fractures and bone loss^(28)^. Considering the effect of nutrients on performance and health, these results reveal that soccer referees need to increase their nutritional knowledge level to provide a healthier diet. In a study^(29)^, most of the athletes stated that the sources of nutritional information were their trainers (25 %) and websites (21 %). Dietitians were at the bottom with 6 %. In the present study, most of the referees did not study nutrition education during the education process and stated the source of nutrition knowledge as TV-internet, and only 19⋅2 % stated the source of nutritional knowledge was dietitians (Table 1). These situations reveal that dieticians should play an active role in nutrition education to increase nutrition knowledge, enabling athletes/soccer referees to make the right food choices in their daily lives.

The Union of European Football Associations (UEFA) soccer referee committee considers diet and physical training. The referee must have good biological support for optimal fitness, which provides motor abilities throughout the match. Besides being crucial for optimal body weight and composition^(30)^, body composition is a factor closely related to sportive performance that helps to evaluate physical characteristics and nutritional behaviour. The FM% for male soccer players is about 10 %. For soccer referees, this ratio is in the range of 11⋅7–13⋅7 %^(31–33)^. In the present study, FM% was 13⋅4 ± 4⋅39 % (Table 2). Muniroglu ve Subak^(34)^ stated that there was no correlation between the 5, 10 and 30 m sprint test and body weight and height. In the present study, there was a statistically significant positive correlation between BMI and 10 and 30 m sprint tests and a statistically significant positive relationship between FM% and 10, 30 and 50 m sprint tests (Table 5). This situation reveals that BMI and FM% are anthropometric measurements that should be taken into account in increasing the performance of soccer referees. Another anthropometric measurement related to performance is WC. In a study, a negative, statistically significant relationship was found between WC, muscle strength and cardiorespiratory fitness/aerobic endurance^(35)^. In the present study, a statistically significant negative was found between WC and the cooper test; a statistically significant positive correlation was found between the 5, 10 and 30-m sprint tests (Table 5). These results reveal that not only BMI but also WC is a parameter that should be taken into consideration in clinical applications and scientific studies.

In athletes, gender, age, current level and competition level affect their anthropometric, physiological and physical characteristics. This situation reveals the necessity of individual nutrition and training programmes to achieve maximum performance. In a systematic review evaluating male footballers, 10 and 30 m sprint speeds, FM% and muscle strength results of higher-level athletes were found to be at a better level than amateur and recreational athletes^(36)^. It was determined that the class referees' 5, 10 and 30 m sprint and cooper test scores and FM% values were at a better level than the referees in the sub-class in this study. For the cooper test, this difference was statistically significant (Table 4). This may be related to the increase of the physical and physiological demands expected of the athlete to stay at those levels and to continue her/his success as the level and competition level of the athlete increases. In addition, the correlation coefficients in sprint tests are higher than those in cooper tests. This may be explained by sprint tests require explosive power, while the cooper test requires long-term endurance.

The use of four different methods to determine athletic performance is the strength of this study. Conversely, our study has some limitations. The sample size was small due to the specific study sample. Also, candidate referees, who are the lowest referee classification, could not be included in the study because they are at the beginning of the refereeing and do not have sufficient training levels.

## Conclusions

There is no miracle food to meet all the needs alone. The ideal approach in this regard is to provide diversity in food choices and a balanced diet that will ensure optimal body composition. This balance must be achieved by taking into account the physical activity performed (e.g. training or match). Nutritional recommendations for referees should be made by nutritionists specific to the individual, taking into consideration gender, age, physical characteristics, body composition, special needs, training intensity and match frequency.
